# Microbial Larvicide Application by a Large-Scale, Community-Based Program Reduces Malaria Infection Prevalence in Urban Dar Es Salaam, Tanzania

**DOI:** 10.1371/journal.pone.0005107

**Published:** 2009-03-31

**Authors:** Yvonne Geissbühler, Khadija Kannady, Prosper Pius Chaki, Basiliana Emidi, Nicodem James Govella, Valeliana Mayagaya, Michael Kiama, Deo Mtasiwa, Hassan Mshinda, Steven William Lindsay, Marcel Tanner, Ulrike Fillinger, Marcia Caldas de Castro, Gerry Francis Killeen

**Affiliations:** 1 Department of Public Health and Epidemiology, Swiss Tropical Institute, Basel, Switzerland; 2 Dar es Salaam City Council, Ministry of Regional Administration and Local Government, Dar es Salaam, United Republic of Tanzania; 3 Coordination Office, Ifakara Health Institute, Dar es Salaam, United Republic of Tanzania; 4 School of Biological and Biomedical Sciences, Durham University, Durham, United Kingdom; 5 Department of Zoology and Marine Biology, University of Dar es Salaam, Dar es Salaam, Tanzania; 6 Ministry of Health and Social Welfare, Dar es Salaam, United Republic of Tanzania; 7 Department of Population and International Health, Harvard School of Public Health, Boston, Massachusetts, United States of America; 8 Vector Group, Liverpool School of Tropical Medicine, Liverpool, United Kingdom; St. George's Hospital Medical School, United Kingdom

## Abstract

**Background:**

Malaria control in Africa is most tractable in urban settlements yet most research has focused on rural settings. Elimination of malaria transmission from urban areas may require larval control strategies that complement adult mosquito control using insecticide-treated nets or houses, particularly where vectors feed outdoors.

**Methods and Findings:**

Microbial larvicide (*Bacillus thuringiensis* var. *israelensis* (*Bti*)) was applied weekly through programmatic, non-randomized community-based, but vertically managed, delivery systems in urban Dar es Salaam, Tanzania. Continuous, randomized cluster sampling of malaria infection prevalence and non-random programmatic surveillance of entomological inoculation rate (EIR) respectively constituted the primary and secondary outcomes surveyed within a population of approximately 612,000 residents in 15 fully urban wards covering 55 km^2^. *Bti* application for one year in 3 of those wards (17 km^2^ with 128,000 residents) reduced crude annual transmission estimates (Relative EIR [95% Confidence Interval] = 0.683 [0.491–0.952], P = 0.024) but program effectiveness peaked between July and September (Relative EIR [CI] = 0.354 [0.193 to 0.650], P = 0.001) when 45% (9/20) of directly observed transmission events occurred. Larviciding reduced malaria infection risk among children ≤5 years of age (OR [CI] = 0.284 [0.101 to 0.801], P = 0.017) and provided protection at least as good as personal use of an insecticide treated net (OR [CI] = 0.764 [0.614–0.951], P = 0.016).

**Conclusions:**

In this context, larviciding reduced malaria prevalence and complemented existing protection provided by insecticide-treated nets. Larviciding may represent a useful option for integrated vector management in Africa, particularly in its rapidly growing urban centres.

## Introduction

Although awareness and support for controlling malaria has increased greatly in recent years, current financial commitments total only 20% of that required [1], [2] and malaria remains a major contributor to the global disease burden [3]. Malaria research and control has traditionally focused on rural areas but it is increasingly recognized that malaria also poses a major problem in urban settings [4]–[6]. Malaria transmission is generally lower in urban areas but improved understanding and evidence–based strategies for controlling urban malaria [4]–[6] are urgently needed because more than 50% of the African population will live in towns or cities by 2030 [7]. Indeed many of the billion people exposed to low-level malaria risk globally, for whom elimination of local transmission exposure is a feasible ambition [8], live in urban settlements across the tropics [6]. Crucially, malaria is considered easier to control or even eliminate in urban centres because lower, more tractable levels of transmission often coincide with higher population densities, better access to health services, social inclusion and institutional capacity [4]–[6].

In Dar es Salaam, the largest city of the United Republic of Tanzania, inhabitants use different protective measures like ceiling boards, window screening, sprays, coils, repellents and insecticide treated nets (ITNs), depending on what they can afford and on their knowledge and perception of risk [9]. Tanzania has emphasized widespread use of ITNs as a priority malaria vector control strategy [10] but recent observations indicate that malaria vectors tend to bite outdoors in Dar es Salaam so ITNs confer less protection than in rural areas [9]. Alternative strategies which reduce larval abundance and hence adult vector populations [11]–[17] may be of great utility in urban areas with high human population densities, particularly those with similarly exophagic vectors. Successes of larval control and integrated vector control programs including environmental management have been clearly recorded in the past but no consistently sustained example remains today [14], [15], [17], [18]. An Urban Malaria Control Program (UMCP) has recently been initiated by the Dar es Salaam City Council in Tanzania as a pilot program to develop sustainable and affordable systems for larval control as part of routine municipal services. Specifically, the UMCP implements the regular application of microbial larvicides (*Bacillus thuringiensis* var. *israelensis (Bti)* and *B. sphaericus (Bs)* [19]–[25]) through community-based, but vertically managed, delivery systems [26], [27].

Here we evaluate the impact of this programmatic system for regular application of microbial larvicides in urban Dar es Salaam [27]. Specifically, we describe the benefits of this program in terms of reduced malaria transmission and infection prevalence, considering local malaria epidemiology and seasonality, as well as the presence of existing malaria control measures such as ITNs, ceiling boards, and window screening and therapeutic drugs.

## Methods

### Study site and experimental design

This study was conducted in Dar es Salaam, the biggest and economically most important city in Tanzania, which is situated on the shores of the Indian Ocean [14]. Dar es Salaam is a city with a well documented history of successful health sector reform and vector control operations [14], [28]–[38]. It covers a total area of 1400 km^2^ with approximately 2.5 million inhabitants [39]. Dar es Salaam is divided into 3 municipalities: Temeke, Ilala and Kinondoni, which together comprise 73 wards. The wards are further subdivided into neighbourhood-sized administrative subunits known as *mitaa* (singular *mtaa*), the Kiswahili word for street. Such neighbourhoods normally compromise between 20 and 100 *mashina* (singular *shina*) or Ten Cell Unit (TCU). The TCU is the smallest subunit of local government in Tanzania, typically including 20–30 houses but some even exceed 100 (Ref. [40]).

**Figure 1:**
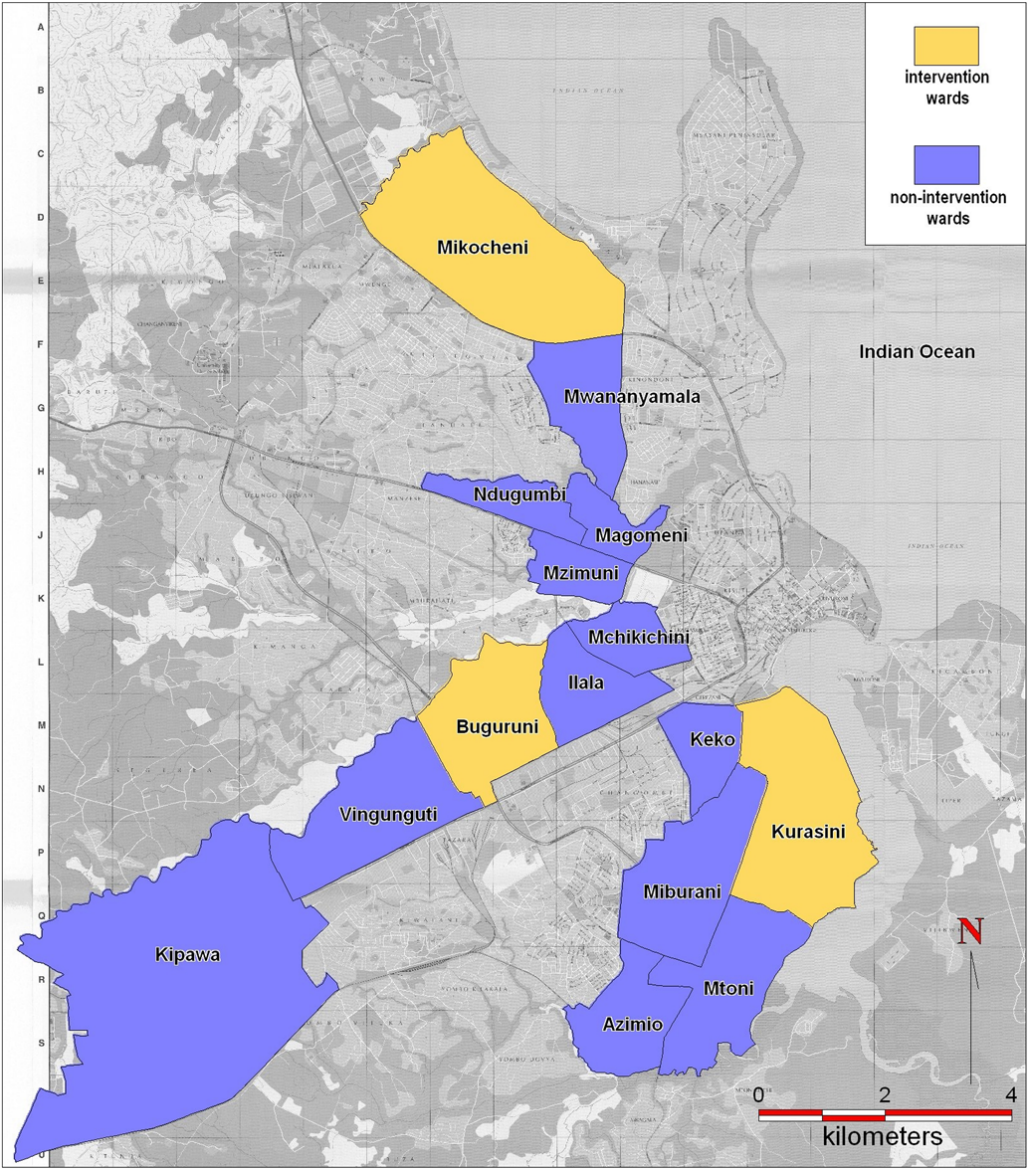
A map of the study area in central Dar es Salaam, Tanzania. Wards included in the study area of the Dar es Salaam Urban Malaria Control Program (UMCP) are outlined, specifying those targeted for larviciding from March 2006 onwards (intervention) and those which did receive any larviciding over the course of the study (non-intervention wards).

**Figure 2:**
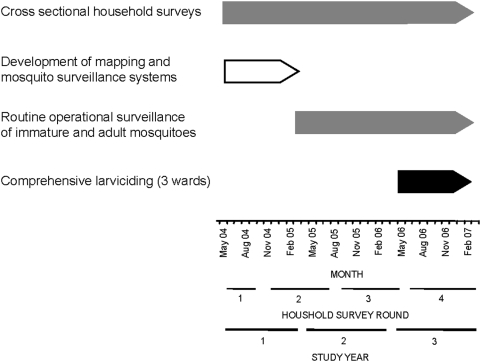
Schematic representation of the timeline of activities described in this study. The study is divided into years of programmatic activity as follows: Year 1: April 2004 till March 2005 was the first year, during which household surveys were initiated and systems for mapping and monitoring larval habitats were developed [27], [40]. Year 2 spans the period April 2005 to March 2006 and was also defined as a pre-intervention year because no larviciding was implemented. In year 2 household surveys were complemented with entomological baseline data (larval and adult surveys) allowing subsequent rational implementation and evaluation of larviciding. Year 3 is the subsequent intervention year during which systematic larviciding was introduced to the three selected wards and spanned the period of April 2006 to March 2007.

**Figure 3:**
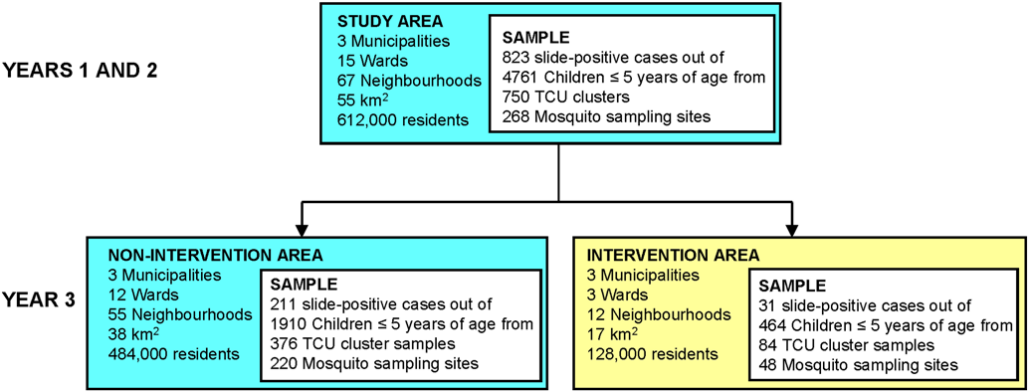
Study area, population, experimental design and sample sizes.

The findings presented here are based on data derived from the first 3 years of the UMCP, where household surveys including malaria infection status were initiated in May 2004. The project area includes 5 wards in each of the three municipalities, comprising a total of 67 *mitaa* (Figure 1). This study site covers a surface area of 55 km^2^ in which 611,871 people resided in 2002 [39]. The new management and delivery systems developed which underpin this program are described in detail elsewhere [27]. The surveillance activities of the UMCP are briefly described below and rely on 3 crucial components: 1) Mapping and surveillance of potential *Anopheles* breeding sites [27], [40], 2) Monitoring of adult mosquito densities [9], [27], and 3) Cluster-sampled household surveys of parasite infection status and potential determinants thereof (Figures 2 and 3). In the third year of the UMCP, beginning in March 2006, the routine application of the microbial larvicide *Bti* to open habitats and *Bs* to closed habitats was initiated in 3 of the 15 wards in the study area [27], adding to existing interventions such as bednets, house screening, ceiling boards, repellents, coils and spray (Figures 1, 2 and 3). Buguruni, Mikocheni and Kurasini wards in Ilala, Kindondoni and Temeke Municipalities, respectively, are home to a total of approximately 128,000 residents and were chosen non-randomly for intervention with larvicides because comprehensive and detailed maps had been completed and larval surveillance teams in these wards were considered most effective [27], [40].

### Programmatic implementation of larvicide application

Larviciding started in March 2006 in one ward of each municipality, namely Buguruni, Mikocheni and Kurasini, encompassing a total surface area of 17 km^2^. These intervention wards were chosen based on the ability of the ward supervisors and the ward-based CORPs to collect, understand, use and submit high quality data during the baseline data collection period [27]. The microbial insecticides applied were *Bacillus thuringiensis* var. *israelensis* (VectoBac®) for open (light-exposed) habitats and *Bacillus sphaericus* (VectoLex®) for closed (covered, often highly polluted) habitats. Open habitats, which have the potential to produce *Anopheles* larvae, were treated weekly by the Mosquito Control CORPs, each of whom was assigned to a specific *mtaa* or portions of an *mtaa*. Open habitats were treated every week with *Bti* at dosages of 0.04 and 1 g per m^2^ for water-dispersible granule and corn cob granule formulations, respectively. Closed habitats, which mainly produce *Culex quinquefaciatus*, were treated every three months by an additional team of CORPs, using *Bs* corn cob granules [27] at a dosage rate of 1 g per m^2^. It should be noted, however, that unlike the open habitats which were the primary focus of this program, no rigorous monitoring and follow up of these closed habitats was implemented.

The primary operational surveillance system used to monitor and manage the application of larvicides was a continuously repeated and comprehensive weekly survey of every potential breeding site in the study area, which included dipping for aquatic-stage mosquito larvae [27]. Before surveillance or control activities started, all active or potential breeding sites in each TCU were recorded by community own resource persons (CORPs) using a set of formalized sketch maps as a rigorous geographic framework that is applicable at community level [40]. Approximately 90 larval surveillance CORPs surveyed all water bodies in their assigned area on a weekly basis for the presence of *Anopheles* and Culicine mosquitoes and report their observations using standardized forms [27].

These larval data are not described in detail here for a number of reasons. Firstly, they were developed as a tool to enable decentralized, daily operational management of larval control at ward level rather than as a rigorous and consistently collected indicator of impact on vector populations. The ward supervisors who collate such data have intrinsic competing interests in reporting shortcomings of control. Secondly, rigorous quality assurance of the larval surveillance was limited to a subset of six wards as previously described [27] so as to enhance control and provide a minimal comparative data set rather than to achieve uniform data quality. Thirdly, larval surveillance is, by definition, biased towards habitats which are already known and therefore most likely to be covered by control activities, so this is an indicator which tends to overestimate impact on mosquito populations. Larval surveillance data are not considered further in this evaluation except as a methodological means for operational teams to monitor and manage control activities.

### Outcome measures

This report focuses on the impact of larviciding on rigorous indicators of malaria transmission and infection risk which are collected completely independently of the teams responsible for control activities so that no biases are introduced. The primary outcome considered here is malaria infection status determined by quality-controlled microscopy of Giemsa-stained thick smears on glass slides, obtained through randomized, cluster-sampled household surveys that also recorded factors which might influence infection risk among these residents. The secondary outcome considered is Entomologic Inoculation Rate (EIR), estimated through routine programmatic entomological surveillance of vector mosquito densities in fixed, non-randomly chosen locations combined with subsequent laboratory analysis of captured specimens for sporozoite-stage parasite infection prevalence. These indicators are not only collected independently of larval control activities, they are also direct measures of actual transmission and therefore capture all local, household and personal variations in all the underlying processes which determine malaria risk.

#### Entomologic Inoculation Rate

In each of the 67 *mitaa*, one resident was recruited as an Adult Mosquito Surveillance CORP in order to conduct human landing catch (HLC) [41]. In each *mtaa*, four different, well-distributed sampling locations were chosen non-randomly in order to maximize coverage of surveillance, resulting in a total of 268 routinely maintained surveillance sites [9]. HLC was conducted once every four weeks at each location outdoors from 6pm to 6am for 45 minutes of each hour, allowing 15 minutes break for rest. Measured biting densities were therefore divided by 0.75 to obtain biting rates for a full hour. In order to estimate the total true exposure experienced both indoors and outdoors by residents, these directly measured outdoor mosquito densities were multiplied by the coefficient of the estimated total true human exposure divided by the estimated total outdoor biting rate obtained from detailed studies of mosquito-human interactions [9]. These coefficients (*Anopheles gambiae*: 0.670, *An. funestus*: 0.725, *An. coustani*: 0.448 and *Culex*: 0.94) were derived from an in-depth behavioural survey of both mosquitoes and humans which was conducted during the main rainy season of April to June 2006 [9]. All mosquitoes were identified morphologically to genus and, in the case of *Anopheles*, to species complex level [42], [43]. Members of the *An. gambiae* complex were further identified to sibling species level by polymerase chain reaction (PCR) [44]. The sporozoite infection status of each mosquito was determined by enzyme-linked immunoabsorbent assay as previously described [45]. Note that adult mosquito surveillance for the entire study area was coordinated centrally and independently of the decentralized larvicide application teams to provide unbiased, uniform and independently collected data for both programmatic monitoring [27] and rigorous evaluation.

Generalized estimating equations (GEE) were used to estimate impact on mosquito densities and EIR by treating active larviciding in that time and place as a categorical independent factor in the model (SPSS® 15.0). Although the first larviciding began in March 2006, these activities took some weeks to scale up across the full extent of the three targeted wards so for analytical purposes we consider March 2006 to be the last month of pre-intervention year 2. Apart from the programmatic rationale for this assumption, biologically-determined time lags in the affected processes suggest that substantial impact upon either adult mosquito density or, even more so, upon malaria infection prevalence cannot be expected any earlier. In all cases, EIR was calculated as the product of the mean biting density of the vector species over the periods in question, multiplied by the mean sporozoite prevalence for that species over the course of the entire year. Total EIR was calculated as the sum of the EIR values for each vector over the period in question. TCU was treated as the unit of geographic location and year as the indicator of time, with vector densities and EIRs estimated as means for each TCU over either the full year or the duration of the July–September dry season when control appeared most effective [27] and almost half of infected mosquitoes were caught (Results). TCU identity uniquely identifies the 268 sampling sites utilized for these surveys and was therefore treated as a subject variable with mosquito density or total EIR as the dependent variable, using a logarithmic link function and normal distribution, weighted according to the number of catcher nights for each location. The repetition of measurements within the same TCU experimental units was accounted for by treating year as a source of first order autoregressive within-subject variance. Note that in this analysis all 12 non-intervention wards were used for comparison with the 3 intervention wards which differs from an earlier report limited to the 3 non-intervention wards for which quality-controlled larval habitat data was available [27].

#### Malaria infection prevalence

Four rounds of cluster-sampled household surveys were conducted, the first of which took place from May until September 2004. The second started in November 2004 and ended in July 2005. Round 3 went from September 2005 till May 2006 and round 4 from July 2006 till March 2007. During each round, 10 new TCUs were randomly sampled in each of the 15 UMCP wards and every consenting and assenting individual in the entire cluster was surveyed as follows, regardless of whether that individual had been surveyed in previous rounds. While approximately 150 fresh TCUs were surveyed in each survey round, the full cohort of TCUs sampled on the first and second rounds were also followed-up longitudinally for the duration of the study by inclusion in subsequent rounds. This sample size was estimated, based on mean TCU population size, to enable detection of a 5% absolute difference in the proportion infected or re-infected, equivalent to ±50% relative risk from a baseline prevalence of 10%, at a significance level of 5% with 80% power.

The household surveys utilized a questionnaire that recorded the following information about the household: (i) geographical identification of the area, (ii) house structure with an emphasis on features that prevent mosquito entry, (iii) information about education, occupation and knowledge about malaria of the head of the household, (iv) assets, expenditures and income sources, (v) anti-malarial measures in use, including specific drugs used in the previous two weeks, and (vi) individual, demographic, behavioural and health related information like sleeping behaviour, travelling habits and treatment-seeking behaviour. All consenting participants also provided finger-pricked blood samples for Giemsa-stained thick and thin smear microscopic examination. The accuracy of these blood smear diagnoses was quality controlled internally as previously described [28]. Individuals who were found to be infected with malaria parasites were then treated with appropriate front-line anti-malarial drugs (until August 2004 it was sulphadoxine-pyrimethamine (Fansidar®) which was subsequently replaced by artesunate-amodiaquine (Maladar®)), retested a week later and, if necessary, referred to hospital for treatment of recrudescent infections.

In order to calculate an asset index as a proxy for the socioeconomic status (SES), we applied principal component analysis (PCA) to the recorded possessions of each household [46]. All potentially protective assets, such as mosquito nets, window screenings and ceiling boards, were excluded as this would have compromised the value of such an index as an independent determinant of malaria risk. All livestock ownership variables were also excluded because only a few people owned animals while ownership of beds and mattresses were excluded because almost all households had them. Factor 1, which was concluded to best reflect the asset index, accounted for 28.6% of the variance (Table S1).

Determinants of malaria infection prevalence were estimated using GEEs treating infection status as the dependent variable with a binary distribution (infected or not infected) and logit link function. Follow-up records of infection status in subjects treated for malaria and then retested a week after therapy were not included in this analysis so the only repeated measures in this data set are for those subjects in the cohort of TCUs followed up every 6 to 9 months over the course of the study. Such re-testing of the same individuals was treated as a repeated measure with first order autoregressive covariance. As individual participants were selected using a cluster sampling approach, the identities of these TCUs were treated as a cluster effect.

Initial evaluation of the effect of age upon the relationship between measured EIR exposure and infection status used a GEE that included the logarithm of the total EIR plus one as a continuous variable in the model and stratified the data by age group. These analyses revealed that prevalence was only positively associated with local exposure levels among young children of five years or less (Box S1). All subsequent analyses to estimate the impact of interventions, such as larviciding and insecticide-treated nets, were therefore restricted to this age group.

Although preliminary analysis examined data from each year or round, these were pooled for the final analysis and survey round was treated as the unit of temporal variation. The final model fit was optimized by forward stepwise selection (exclusion criterion; P>0.10) of all potential determinants of malaria risk, such as socioeconomic status and protective measures like coils, sprays, repellents and recent drug use. Initial attempts to fit the model to the entire 7135 human subjects controlling for cluster effects were confounded by the covariance between time, location and intervention which occurred because many TCUs were only surveyed either before or after the introduction of larviciding. Although treating the TCU as the experimental unit overcame this problem and allowed the impact of larviciding to be estimated, this approach precluded the detection of important risk factors operating at the individual level, such as personal use of insecticide-treated nets. We therefore confirmed this estimate while controlling for individual-level factors by treating individual human subject as the experimental unit and preventing TCU-larviciding covariance by restricting our analysis to the 4450 individuals from TCUs which were surveyed at least once before and once after larviciding was introduced to three of the 15 wards.

### Ethics Statement

All activities of the UMCP, including these field surveys were approved by the Medical Research Coordination Committee of the National Institute for Medical Research, Ministry of Health, Government of Tanzania (Reference numbers NIMR/HQ/R.8a/Vol. IX/279 and 324) and Durham University's Ethics Advisory Committee. No persons in high risk groups, namely people under 18 years or women of reproductive age, were recruited to conduct human landing catch. Furthermore, all human landing catchers were screened weekly for malaria by microscopic examination of thick smear peripheral blood samples and, when found infected, treated with artemisin-based combination therapy. Participating residents in the household survey signed an informed consent form after receiving information about the goal, objectives, risks and benefits of the study. For children under 18 years, parents or designated guardians granted consent. Individual information was kept in strict confidence by storing in locked rooms and cabinets and password-protected computers.

## Results

### Mosquito densities, malaria prevalence and seasonality

All three species of *Anopheles* we recorded in Dar es Salaam, namely *An. gambiae s.l.*, *An. funestus* and *An. coustani*, were identified as malaria vectors but their generally low densities and sporozoite infection prevalence resulted in relatively modest transmission intensity of just over 1 infectious bite per year (Table 1). Correspondingly, the prevalence of malaria infection amongst humans was also moderate: between May 2004 and March 2007 the crude prevalence of malaria infection across all age groups averaged 11.7% (4969/42,447) but steadily declined from 17.6% in year 1 (2189/12,431) to 11.9% (1614/13,563) in year 2 and 7.1% (1166/16,453) in year 3. As described in Box S1, the age-prevalence profile indicated substantial levels of immunity, and therefore historical exposure, among older age groups.

**Table 1:** Crude entomological estimates of mosquito density and malaria transmission in Dar es Salaam.

Parameter	*An. gambiae*	*An. funestus*	*An. coustani*	All Anopheles	*Culex* sp.
Total mosquitoes caught	3868	160	936	4964	240295
Estimated mean biting rate (bites per person per night)^a^	0.63	0.03	0.10	0.76	55.1
Number sporozoite positive	16	2	5	23	NA
Sporozoite prevalence (%)	0.41	1.25	0.53	0.46	NA
Entomological inoculation rate (infectious bites per person per year)^b^	0.95	0.13	0.20	1.28	NA

The parameters presented are directly derived from or estimated from a total of 5463 nights of human landing catches distributed across the UMCP area (Figure 1) in years 2 and 3 of the study (Figure 2).

a Total number caught×(species-specific quotient of mean overall exposure / mean outdoor biting rate as per reference [9]) / (0.75× total catcher nights).

b Estimated biting rate ×365 days per year×mean sporozoite prevalence.

**Figure 4:**
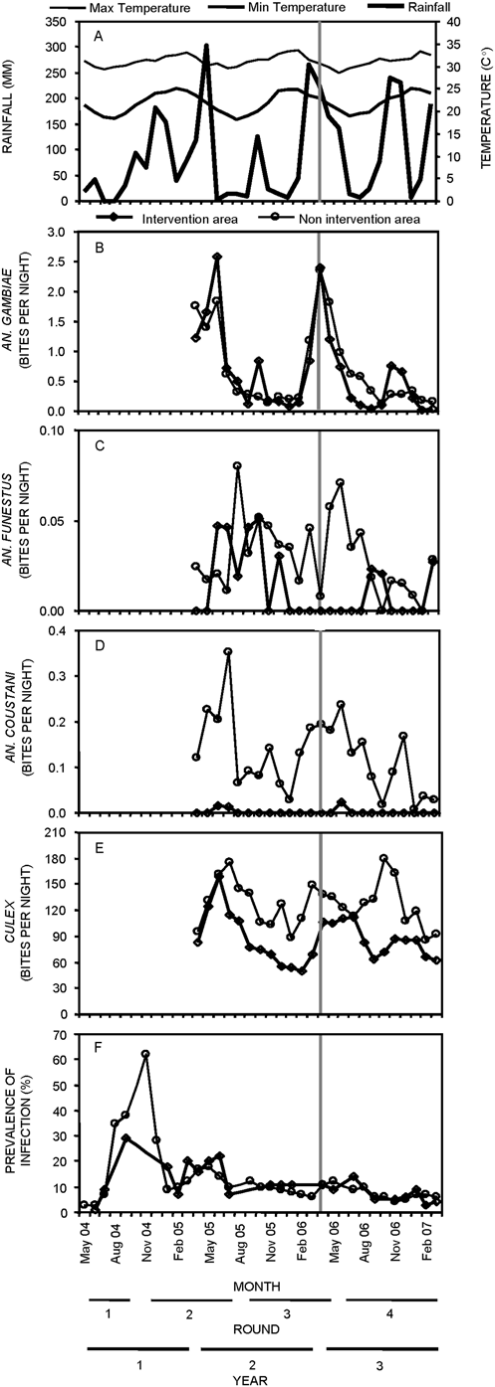
Longitudinal trends in overall malaria prevalence and its environmental determinants in Dar es Salaam, Tanzania. Monthly variations in rainfall, temperature (A), mosquito biting densities (B–E) and malaria prevalence (F) in the intervention and non-intervention areas over the first three years of the urban malaria control program (UMCP). Climatic and prevalence data was available from May 2004 till March 2007 whereas mosquito data was only collected from April 2005 till March 2007. Meteorological data was derived from meteorological station at Nyerere International Airport and assumed representative of both intervention and non-intervention areas.

**Figure 5:**
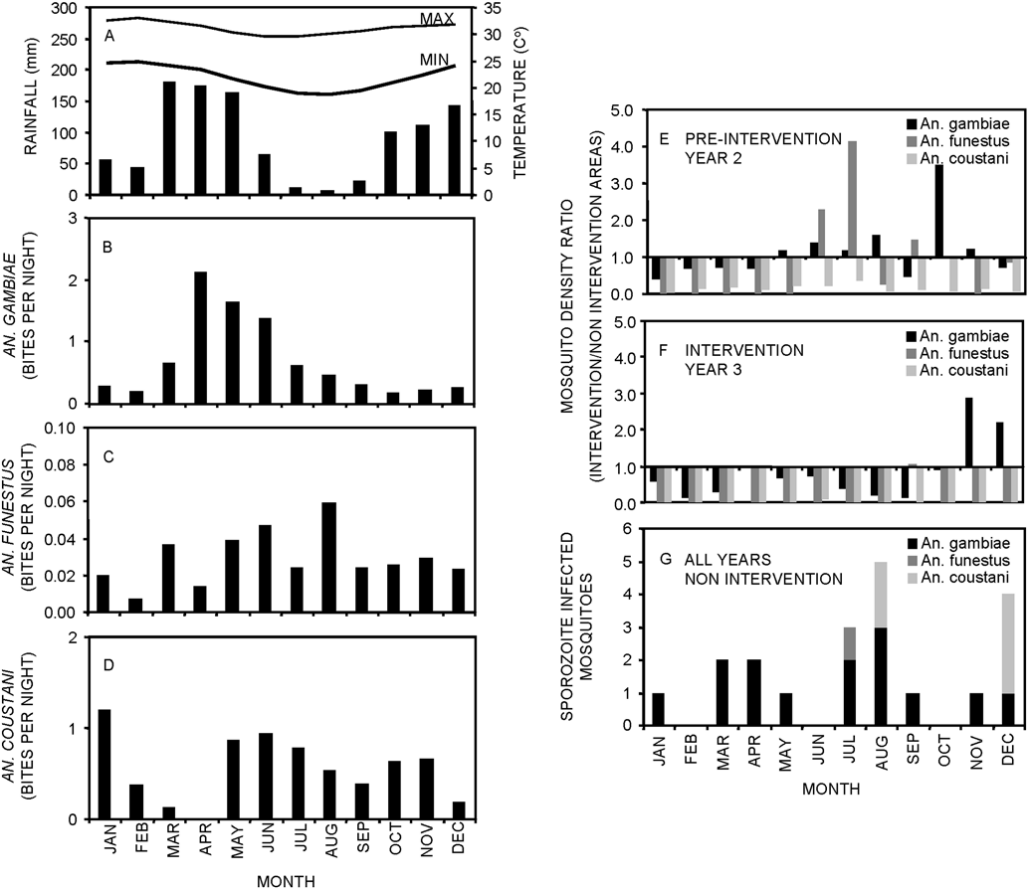
Seasonal patterns of malaria transmission in Dar es Salaam, Tanzania. Seasonal patterns of rainfall and temperature (A), seasonal distribution of mosquito biting densities (B–D) and sporozoite-infected mosquitoes in the non-intervention areas (G), as well as relative biting rates in the pre-intervention and the intervention year (E, F). Relative biting densities were aggregated over pre-intervention year 2 (E: April 2004 till March 2005) and intervention year 3 (F: April 2005 till March 2006)) while direct observations of transmission in the non-intervention areas (G) were summed over both years to consolidate the limited numbers of observations in a qualitatively useful manner.

Mosquito abundance and malaria prevalence followed seasonal patterns in Dar es Salaam (Figure 4 and 5). Peak *An. gambiae s.l.* densities occurred shortly after the peak of the main rains in April–May (Figure 4B and 5B), whilst *An. funestus* had a much longer time lag, peaking around July and August (Figure 4C and 5C). *An. coustani* densities were highest in January shortly after the short rainy season, following which they almost disappear, reappearing and persisting immediately after the main rains. Note, however, that *An. coustani* densities in the intervention areas were generally very low, only appearing in June and July (Figure 4D and 5D). *Culex sp.* densities were also highest during and shortly after the main rainy season (Figure 4E). Interestingly, malaria prevalence peaked at different times each year (Figure 4F). In 2004, prevalence reached extremely high levels in November, appearing to reflect an active epidemic. Epidemic-prone conditions may have resulted from the low prevalence and immunity levels experienced during the exceptionally dry periods in 2003 and early 2004 [28]. In both 2005 and 2006 there was a clear peak of infection prevalence in or around May (Figure 4F).

### Impact of larvicides upon mosquito densities and malaria transmission

Larviciding substantially suppressed annual mean densities of both secondary vectors in Dar es Salaam, namely *An. funestus* and *An. coustani* (Table 2). Although no significant suppression of the primary vector *An. gambiae* was observed over the course of the year, total EIR calculated from the combined annual mean densities and sporozoite prevalences of all three malaria vectors was reduced by 32% (Table 2). Densities of *Culex* sp. were only slightly reduced so it is unlikely that any worthwhile suppression of biting nuisance or transmission of other pathogens was achieved (Table 2).

**Table 2:** Impact of larviciding on mosquito density and crude malaria transmission intensity^a^ in year 3 of the study estimated using generalized estimating equations as described in the methods section (n = 268 mosquito sampling sites which were non-randomly assigned to 4 fixed TCUs per neighbourhood and monitored throughout the second and third years of the study).

Parameter	Relative biting or transmission intensity [95% CI]	P-value
Full year		
*An. gambiae*	0.769 [0.551–1.073]	0.123
*An. funestus*	0.342 [0.113–1.039]	0.058
***An. coustani***	**0.020 [0.003–0.149]**	**<0.001**
**Total EIR**	**0.683 [0.491–0.952]**	**0.024**
***Culex*** ** sp.**	**0.862 [0.756–0.983]**	**0.027**
July–September		
***An. gambiae***	**0.278 [0.145–0.531]**	**<0.001**
*An. funestus*	0.270 [0.052–1.41]	0.121
*An. coustani*	0.926 [0.799–1.023]	0.306
**Total EIR**	**0.354 [0.193–0.650]**	**0.001**
***Culex*** ** sp.**	**0.790 [0.672–0.928]**	**0.004**

a Annual or seasonal mean biting rate×mean annual sporozoite prevalence.

The modest and non-significant impact of larviciding upon annual mean densities of *An. gambiae* (Figure 4B, Table 2) is consistent with analyses restricted to 6 of the study wards from which quality-controlled larval data was available [27]. However, it is noteworthy that *An. gambiae* was controlled most effectively during drier periods while control during the two wet periods of year 3 was generally poor [27]. Inadequate control during the main rainy season was primarily due to cash flow restrictions which delayed procurement so that larviciding did not begin early enough to prevent the bulk of *An. gambiae* proliferation [27]. Control effectiveness also relapsed during the short rains due to newly generated, inaccessible larval habitats in waste water settlement ponds [27]. Taken at face value, such an apparently modest overall reduction of transmission would not be expected to yield major public health benefits [5], [6], [47], [48]. However, these conventional analyses are based on mean sporozoite prevalence rates which are assumed to be constant throughout year. This approach fails to capture a more encouraging feature of the data: seasonal coincidence of optimal control effectiveness with the peak of actual transmission rather than with mosquito densities.

Effectiveness of *An. gambiae* control varied seasonally and previous analyses have shown that reduction of *An. gambiae* densities [27] was greatest during the dry season following the main rains (Figure 5E and 5F). Although some sporozoite-infected *An. gambiae* were caught when their abundance peaked in April and May, most were caught during drier months following the cold season when warmer conditions allowed faster parasite development and higher mosquito survival. In fact, almost half of all directly observed transmission events occurred between July and September (Figure 5G) when control of all three confirmed vectors, including *An. gambiae*, was most effective (Figure 5E and 4F): 45% (9/20) of all sporozoite-infected mosquitoes caught in the 12 non-intervention wards occurred in this three month period (Figure 5G). The ratio of *An. gambiae* biting densities for intervention versus non-intervention areas was particularly reduced by larviciding in July and August of year 3 compared to the same period of the pre-intervention year 2 (Figure 5E and 5F). Furthermore, the density ratio of both *An. funestus* and *An. coustani*, which are responsible for about a quarter of all transmission, were greatly reduced throughout the whole intervention year (Table 2, Figure 5E and 4F and Table S2). Consistent with previous analyses restricted to 6 of the study wards, analyses of mosquito densities over the July to September period (Table 2) reveal useful reductions of *An. gambiae* densities (−72%) and malaria transmission (−65%). Thus the seasonal peak in control effectiveness coincided optimally with the period when the vector population was most infectious to humans. Unfortunately, a mere 23 sporozoite-infected mosquitoes are insufficient to estimate the impact of larviciding upon directly measured transmission events by Poisson regression [49]. It is nevertheless encouraging to qualitatively observe fortuitous temporal targeting of effective control of the primary malaria vector, complemented by successful all-year-round abatement of the secondary vectors.

### Impact of larvicides and personal protection measures upon malaria infection prevalence

**Figure 6:**
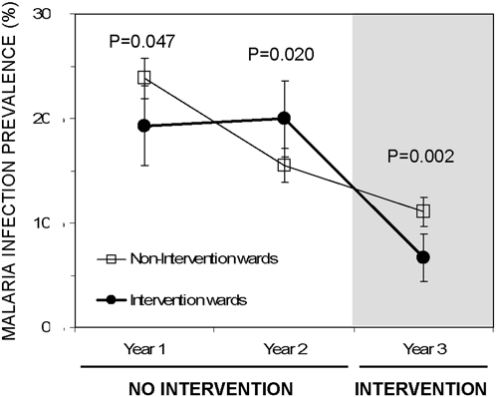
Crude prevalence of malaria infection amongst children of five years or less in intervention and non-intervention wards over each year of the study. Error bars represent 95% confidence intervals with the significance of the differences between intervention and non-intervention wards estimated by χ^2^ analysis (n = 1908, 1983 and 1910 for non-intervention and 414, 456 and 456 for intervention wards in years 1, 2 and 3, respectively).

Initial examination of figure 4F suggests that larviciding had little impact, if any, upon malaria prevalence among all age groups. The observation that infection prevalence and responsiveness to exposure was concentrated in young children (Box S1) prompted us to restrict our analysis of determinants of malaria risk to this youngest age stratum. Crude analysis of prevalence within this age stratum tentatively suggests some benefit of larviciding: Although modest and inconsistent differences do exist between the intervention and non-intervention wards in years 1 and 2, prevalence appears more clearly and significantly lower in the former during year 3 when larviciding was applied (Figure 6). Given that the spatial aggregation of malaria risk is particularly acute in cities [4], [5] and a cluster sampling procedure was used, with different sets of TCUs sampled in each survey round, it is perhaps unsurprising that the crude relationship between intervention and non-intervention areas before the actual introduction of larviciding was slightly inconsistent (Figure 6).

Logistic regression analysis of data from each year revealed that when the effects of cluster sampling was taken into account (Table S3), malaria prevalence in the intervention and non-intervention areas was more readily comparable in years 1 and 2 and that the only significant determinant of infection risk in any year was being in an area with larviciding during year 3 (OR [95% CI] = 0.536 [0.361 to 0.796]). In order to clearly resolve spatial and temporal variation in malaria risk from the impact of larviciding which was delivered to specific geographic areas at specific times, infection status and questionnaire data for all three years were analysed treating survey round and TCU as units of temporal and spatial variation, respectively. Preliminary analyses which also consider TCU as the experimental unit indicated an impressive protective effect of larviciding (OR = 0.313 [0.118–0.830], P = 0.019) and this observation was confirmed by individual-level analyses which also reveal the benefits of selected personal protection measures (Table 3). Apart from survey round and location, the only other factors which even approached significance as determinants of malaria risk were all interventions which prevent exposure to mosquitoes (Table 3). Use of a topical repellent may provide considerable protection and the benefits of either using an insecticide-treated net (ITN) or living in an area which is treated with larvicide are clearly significant. While this individual-level analysis does not capture the mass effect of ITNs upon malaria transmission [50], it is notable that larviciding appears to offer protection against malaria that is at least comparable with actually using an ITN and may well be better (Table 3).

**Table 3:** Impact of larviciding on malaria infection prevalence in children up to five years of age, as determined using generalized estimating equations (n = 4450 individuals from 283 TCUs sampled over the three years of the study).

Parameter	OR [95% CI]	P-value
*Included in the model*		
Location (Ten Cell Unit)	NP	<0.001
Survey Round	NP	<0.001
Larviciding	0.284 [0.101–0.801]	0.017
Insecticide-treated net	0.764 [0.614–0.951]	0.016
Repellent	0.563 [0.286–1.107]	0.096
*Not included in the model*		
Socio-economic status		0.258
Education level		0.529
Anti-malarial drug use in the previous two weeks		0.768
Sleep elsewhere		0.357
First or subsequent survey of individual		0.835
Ceiling board		0.331
Any net		0.973
Window screening		0.881
Coil		0.231
Spray		0.483

NP = Not presented as values for each level are both trivial and numerous.

Modest increases in the use of effective drugs, perhaps combined with increasing use of screening and complete ceilings, may well have played a role in the overall reduction of malaria prevalence over these three years (Box S2). Significant differences were observed in the usage of different control measures in the sampled households of the intervention versus non-intervention areas (Table S4) but none of these differences were consistent with, or of a sufficient magnitude to plausibly explain, the massive reduction of malaria risk in the intervention wards during year 3 (Table S4). The usage rates of personal protection measures, namely ITNs, ceilings and screening differed significantly between intervention and non-intervention wards in year 3 but the magnitude of these differences was very slight and, with the exception of ceilings, would bias towards underestimation of the impact of larviciding (Table S4). Although there were significant variations in proportional use of different types of drugs, the absolute number of individuals was small (Table S4): Only 16% of surveyed individuals had recently used antimalarials and no statistically significant influence upon risk was detected (Table 3, Table S3).

## Discussion

It is well established that *Bti* effectively kills malaria vector mosquito larvae under field conditions in Africa [23], [25], [51] and can reduce malaria vector population densities [19]–[25], [27]. The impact upon malaria disease burden of microbial larvicides and other forms of larval control against African malaria vectors has been demonstrated in qualitative terms [11]–[15], [17] by historical programs, all of which predate modern standards of rigorous evaluation. More recent efficacy trials in the rural highlands of Western Kenya illustrate that microbial larvicide application protects against malaria infection when delivered as a supplementary measure alongside ITNs [52]. Here we demonstrate the effectiveness of a large-scale, community-based but vertically managed operational program using *Bti* in sub-Saharan Africa in terms of reduced malaria infection prevalence.

Community-based larval control with *Bti*, delivered using the novel management and delivery systems developed by the UMCP [27], [40] reduced malaria prevalence in this setting, suggesting that such approaches might be useful in towns and cities elsewhere in Africa. At an annual cost of approximately US$0.94 per person protected [53], the routine application of larvicides in Dar es Salaam, compares well with the US$1.48 to US$2.64 estimated per year of protection for a long lasting ITN [54] although it should be remembered that the latter often protects more than one person. It is particularly notable that the application of larvicides provides protection which is at least as good as personal use an ITN in both urban Dar es Salaam (Table 3) and the rural highlands of western Kenya[52]. Neither study captures the communal effects of ITNs, which can be just as important as personal protection [50]. However, such area-wide effects require the combined contributions of personal protection by a large proportion of individuals within a population [50]. It is therefore worrying that such a modest individual-level benefit of ITN use was detected in Dar es Salaam (Table 3), particularly when malaria vectors in this context have recently been observed to feed outdoors more frequently than their rural counterparts and, in the case of *An. arabiensis*, feed early in the evening before people go indoors [9]. Larviciding may therefore be at least as cost-effective as ITNs in cities and merits consideration for broader development and evaluation in urban Africa.

A crucial point we emphasize is that the benefits of larviciding are complementary to those of ITNs and no evidence is presented here, or in the recent report from rural Kenya [52], that larviciding represents an alternative to the current front-line vector control tools, namely indoor residual spraying or ITNs. Although the numerical estimates of protection are greater for larvicides than for personal use of ITNs, particularly in Dar es Salaam, these differences are not significant and ignore the area-wide benefits of widespread net use. We therefore suggest that larviciding should be viewed as a supplementary means to control or even malaria eliminate malaria transmission as a component of integrated vector management packages which are rationally adapted to specific settings where it may be an appropriate option. ITNs and indoor residual spraying are, and should remain, the highest priority and most broadly applicable front-line malaria vector control measures. These personal and household protections measures may be augmented with, but not replaced by, larval control where and when it is likely to be feasible and effective. It remains to be determined what those criteria for inclusion of larval control into integrated vector management packages may be. A considerable body of research remains to be completed before the full potential and limitations of larval control are adequately understood.

While these results, and those from a recent trial in rural Kenya [52], are encouraging, this study has substantial limitations. At this early stage, these two reports merely re-open the subject of larval control as an option for discussion. Further studies, with larger sample sizes distributed across larger, more heterogeneous populations in a diversity of settings will be required to determine whether these results are reproducible and broadly applicable. The substantial but nevertheless limited scale of this evaluation in only one geographic location, with non-random assignment of the intervention, using one implementation model over only one year means that this report raises far more questions than it answers. It remains to be seen whether larval control can be sustained effectively in Dar es Salaam, the highlands of Kenya [52] or elsewhere in Africa. If larvicides do indeed have a place in the repertoire of local and national malaria control programs of the future, optimal models for funding, governance, implementation, monitoring and evaluation remain to be elucidated. Nevertheless, these encouraging recent results from both an urban and a rural African context [52] may be very useful in that they prompt re-opening of a discussion which has been considered closed for decades [15], [55].

The observation that larval control can have such clear benefits, even when applied sub-optimally, is surprising and questions the assumption that very high levels of programmatic performance are essential for this approach to deliver epidemiological impact [55], [56]. The surprisingly dramatic impact of larviciding upon malaria prevalence supports the view that actual reductions of transmission in Dar es Salaam were far greater than those obtained from crude estimates based annual mean vector densities and sporozoite prevalence levels (Table 2) because of the fortuitous seasonal interaction of transmission intensity and program effectiveness. This point is further reinforced when one considers that ITN use reduces exposure to malaria vectors by approximately 59% in Dar es Salaam [9] yet the measured protection against blood-stage infection which larviciding provides appears to be as good as this front-line vector control measure and may even be substantially better (Table 3). However, we anticipate that even greater impacts can be achieved as the proficiency of operational teams matures through direct experience and innovation in response to locally-specific operational challenges, as well as improved institutional and financing mechanisms [27]. Tactically, we emphasize the specific need to tackle malaria vector populations in Dar es Salaam more effectively during the long rains while building upon successes during drier times of the year when much transmission occurs but larval habitats are both less abundant and easier to access. Strategically, we conclude that larviciding may well have potential for sustainable malaria control in African cities but emphasize that the encouraging results presented here merely represent an early demonstration which prompts more extensive and intensive investigation of this long-neglected approach.

### Conclusions

Recent results from the rural highlands of western Kenya demonstrate that well-implemented larval control can be highly efficacious as a component of an integrated vector management package which also includes ITNs [52]. Here we demonstrate that similar levels of protective effectiveness can be achieved under routine, real-world programmatic conditions in a major African city. The community-based larval control program we evaluated in Dar es Salaam applied *Bti* on a substantial operational scale (128,000 residents protected) to achieve a dramatic reduction of malaria prevalence. As the last programmatic successes of larval control rapidly fade from living memory [15]–[17], [55], perhaps it is time to re-examine the theoretical considerations [57], [58] which led to half a century of exclusive emphasis upon adult mosquito control for malaria prevention in Africa and beyond [15], [17]. We conclude that larval control should now be reconsidered as an option for integrated malaria control programs in Africa. An immediate priority is to evaluate larval control strategies in further rigor over the long term, particularly in urban areas where feasibility and cost-benefit ratio may be highest. We caution that the evidence base supporting larval control as an intervention option for malaria prevention in Africa remains grossly underdeveloped and needs to be strengthened. We nevertheless suggest that the encouraging early results reported here may be improved upon with time, investment, experience and creativity.

## Supporting Information

Table S1 Asset ownership for households in each socioeconomic status quintile.
(0.04 MB DOC)

Table S2 Comparison of mosquito densities, combined crude indirect EIR of An. gambiae, An. funestus and An. coustani in the intervention and non-intervention area in the two years of the entomological survey. Mosquito survey started in year 2 (April 2005–March 2006) and the intervention with larvicide (Bti) started in year 3 (April 2006–March 2007).
(0.03 MB DOC)

Table S3 The influence of various protection measures and vector control activities upon malaria infection risk amongst children ≤5 in each of years 1 (May 2004–March 2005) and 2 (April 2005–March 2006) of the study before intervention, as well as in year 3 (April 2006–March 2007) during which larviciding was implemented in the three selected wards.
(0.04 MB DOC)

Table S4 Comparison of protective measures and drug use in intervention and non-intervention areas of the Urban Malaria Control Program. Usage of different protection measures and drugs in intervention and non-intervention area was compared by χ2 for each year. The application of larvicide (Bti) started beginning of year 3.
(0.05 MB DOC)

Box S1 Malaria prevalence as a function of age and exposure.
(0.16 MB DOC)

Box S2 Contributions of interventions other than larviciding to malaria prevention.
(0.05 MB DOC)
