# Supplementary material for: Microbial Larvicide Application by a Large-Scale, Community-Based Program Reduces Malaria Infection Prevalence in Urban Dar Es Salaam, Tanzania
Source: PLoS One. 2009 Mar 31;4(3):e5107. doi: 10.1371/journal.pone.0005107 (PMC2661378; doi:10.1371/journal.pone.0005107)
Supplement: Table S1 — Asset ownership for households in each socioeconomic status quintile. (0.04 MB DOC) [file pone.0005107.s001.doc]

**Table S1.** Asset ownership for households in each socioeconomic status quintile

|  | SES quintiles | |  |  |  |
| --- | --- | --- | --- | --- | --- |
|  | Poorest | Very poor | Poor | Less poor | Least poor |
| Clothing cupboard (%) | 15 | 20 | 93 | 97 | 99 |
| Sofa set (%) | 31 | 96 | 99 | 100 | 100 |
| Watch/ clock (%) | 33 | 91 | 97 | 98 | 100 |
| Iron (%) | 27 | 84 | 98 | 99 | 100 |
| Radio (%) | 69 | 99 | 100 | 100 | 100 |
| Bicycle (%) | 3 | 5 | 7 | 10 | 18 |
| Motorcycle (%) | 0 | 0 | 0 | 1 | 8 |
| Car / tractor (%) | 0 | 0 | 0 | 1 | 16 |
| TV (%) | 2 | 10 | 25 | 72 | 87 |
| Satellite dish (%) | 0 | 0 | 0 | 0 | 8 |
| Fan (%) | 4 | 11 | 24 | 75 | 93 |
| Sewing machine (%) | 1 | 2 | 4 | 11 | 31 |
| Video (%) | 0 | 3 | 10 | 40 | 84 |
| CD player (%) | 0 | 2 | 6 | 61 | 96 |
| Camera (%) | 0 | 0 | 0 | 1 | 36 |
| Telephone (%) | 0 | 0 | 0 | 1 | 26 |
| Refrigerator (%) | 5 | 9 | 28 | 66 | 74 |
