# Supplementary material for: Microbial Larvicide Application by a Large-Scale, Community-Based Program Reduces Malaria Infection Prevalence in Urban Dar Es Salaam, Tanzania
Source: PLoS One. 2009 Mar 31;4(3):e5107. doi: 10.1371/journal.pone.0005107 (PMC2661378; doi:10.1371/journal.pone.0005107)
Supplement: Table S2 — Comparison of mosquito densities, combined crude indirect EIR of An. gambiae, An. funestus and An. coustani in the intervention and non-intervention area in the two years of the entomological survey. Mosquito survey started in year 2 (April 2005–March 2006) and the intervention with larvicide (Bti) started in year 3 (April 2006–March 2007). (0.03 MB DOC) [file pone.0005107.s002.doc]

**Table S2.** Comparison of mosquito densities, combined crude indirect EIR of *An. gambiae, An. funestus* and *An. coustani* in the intervention and non-intervention area in the two years of the entomological survey. Mosquito survey started in year 2 (April 2005 – March 2006) and the intervention with larvicide (*Bti*) started in year 3 (April 2006 – March 2007).

| Variables | Year 2 | | | Year 3 | | |
| --- | --- | --- | --- | --- | --- | --- |
|  | Non-intervention area | Intervention area |  | Non-intervention area | Intervention area |  |
|  | Mean [95% CI] | Mean [95% CI] | P | Mean [95% CI] | Mean [95% CI] | P |
| *An. gambiae* (bites per night) | 0.664 [0.538 – 0.819] | 0.719 [0.507 – 1.019] | 0.702 | 0.614 [0.482 – 0.783] | 0.513 [0.383 – 0.686] | 0.350 |
| *An. funestus* (bites per night) | 0.036 [0.021 – 0.061] | 0.021 [0.007 – 0.065] | 0.400 | **0.026 [0.012 – 0.057]** | **0.005 [0.002 – 0.016]** | **0.021** |
| *An. coustani* (bites per night) | **0.143 [0.077 – 0.267]** | **0.003 [0.001 – 0.010]** | **< 0.001** | **0.103 [0.059 – 0.178]** | **0.002 [0.0003 – 0.015]** | **< 0.001** |
| Total EIR (infectious bites per year) | 1.435 [1.137 – 1.813] | 1.178 [0.804 – 1.725] | 0.386 | **1.236 [0.974 – 1.568]** | **0.796 [0.599 – 1.059]** | **0.020** |
| *Culex* (bites per night) | **130 [115 – 146]** | **87 [73 – 104]** | **< 0.001** | **126 [112 – 143]** | **86 [71 – 104]** | **0.001** |

Generalized estimating equations (GEE) was used with TCU as a subject unit, log linked mosquito densities which were weighted by number of catcher nights as a dependent and intervention and non-intervention as a factor.
