# Supplementary material for: Microbial Larvicide Application by a Large-Scale, Community-Based Program Reduces Malaria Infection Prevalence in Urban Dar Es Salaam, Tanzania
Source: PLoS One. 2009 Mar 31;4(3):e5107. doi: 10.1371/journal.pone.0005107 (PMC2661378; doi:10.1371/journal.pone.0005107)
Supplement: Table S3 — The influence of various protection measures and vector control activities upon malaria infection risk amongst children ≤5 in each of years 1 (May 2004–March 2005) and 2 (April 2005–March 2006) of the study before intervention, as well as in year 3 (April 2006–March 2007) during which larviciding was implemented in the three selected wards. (0.04 MB DOC) [file pone.0005107.s003.doc]

**Table S3.** The influence of various protection measures and vector control activities upon malaria infection risk amongst children ≤5 in each of years 1 (May 2004 – March 2005) and 2 (April 2005 – March 2006) of the study before intervention, as well as in year 3 (April 2006 – March 2007) during which larviciding was implemented in the three selected wards.a

| Parameter | P-value | | |
| --- | --- | --- | --- |
|  | Year 1  (n=2322) | Year 2  (n=2439) | Year 3  (n=2374) |
| Larviciding area | 0.369 | 0.057 | **0.002** |
| Socio-economic status | 0.231 | 0.933 | 0.075 |
| Education level | ND | 0.354 | 0.196 |
| Any net | 0.609 | 0.513 | 0.674 |
| Insecticide-treated net | 0.187 | 0.174 | 0.373 |
| Repellent | ND | 0.874 | 0.497 |
| Recent anti-malarial drug use | 0.447 | 0.689 | 0.600 |
| Sleep elsewhere | 0.101 | 0.694 | 0.051 |
| First or subsequent survey of individual | 0.090 | 0.576 | 0.599 |
| Window screening^b^ | 0.204 | 0.996 | 0.269 |
| Ceiling board^c^ | 0.262 | 0.106 | 0.889 |
| Coil | 0.163 | 0.696 | 0.644 |
| Spray | 0.604 | 0.443 | 0.584 |

^a^ Determined using generalized estimating equations (GEE) as described in the main text as restricting analysis to one study year at a time. Effects of survey round and TCU not shown (P<0.001 for both).

^b^ Complete screening, screening with small holes, glass windows versus no screening or badly damaged screening

^c^ Complete and partly complete ceiling board versus no ceiling board

ND: Not determined
