# Supplementary material for: Microbial Larvicide Application by a Large-Scale, Community-Based Program Reduces Malaria Infection Prevalence in Urban Dar Es Salaam, Tanzania
Source: PLoS One. 2009 Mar 31;4(3):e5107. doi: 10.1371/journal.pone.0005107 (PMC2661378; doi:10.1371/journal.pone.0005107)
Supplement: Table S4 — Comparison of protective measures and drug use in intervention and non-intervention areas of the Urban Malaria Control Program. Usage of different protection measures and drugs in intervention and non-intervention area was compared by χ2 for each year. The application of larvicide (Bti) started beginning of year 3. (0.05 MB DOC) [file pone.0005107.s004.doc]

**Table S4.** Comparison of protective measures and drug use in intervention and non-intervention areas of the Urban Malaria Control Program. Usage of different protection measures and drugs in intervention and non-intervention area was compared by χ^2^ for each year. The application of larvicide (*Bti)* started beginning of year 3.

|  | Year 1 |  |  |  |  |  | Year2 |  | |  |  | |  | |  | | Year 3 | |  |  |  | |  | | |
| --- | --- | --- | --- | --- | --- | --- | --- | --- | --- | --- | --- | --- | --- | --- | --- | --- | --- | --- | --- | --- | --- | --- | --- | --- | --- |
| Variables | Non-intervention area | | Intervention area | | | | Non-intervention area | | | Intervention area | | | | | | | Non-intervention area | | Intervention area | | | | | | |
|  | n/N | % | n/N | % | χ^2^ | P | n/N | | % | n/N | | % | | χ^2^ | | P | n/N | % | n/N | | | % | | χ^2^ | P |
| Personal protection |  |  |  |  |  |  |  | |  |  | |  | |  | |  |  |  |  | | |  | |  |  |
| ITN | 2481/10507 | 23.6 | 479/2058 | 23.3 | 1.0 | 0.741 | 3026/10932 | | 27.7 | 708/2695 | | 26.3 | | 0.9 | | 0.142 | **3237/13137** | **24.6** | **732/3264** | | | **22.4** | | **0.9** | **0.008** |
| Window screening |  |  |  |  |  |  |  | |  |  | |  | |  | |  |  |  |  | | |  | |  |  |
| Complete^a^ | 758/1252 | 60.5 | 163/270 | 60.4 | 1.0 | 0.946 | **537/811** | | **66.2** | **255/325** | | **78.5** | | **1.9** | | **< 0.001** | **1394/1834** | **76.0** | **462/527** | | | **87.7** | | **2.2** | **< 0.001** |
| Ceiling board |  |  |  |  |  |  |  | |  |  | |  | |  | |  |  |  |  | | |  | |  |  |
| Complete^b^ | 526/1252 | 42.0 | 101/271 | 37.3 | 0.8 | 0.151 | **406/811** | | **50.1** | **186/326** | | **57.1** | | **1.3** | | **0.033** | **1141/1834** | **62.2** | **289/527** | | | **54.8** | | **0.7** | **0.002** |
| Drug use |  |  |  |  |  |  |  | |  |  | |  | |  | |  |  |  |  | | |  | |  |  |
| Chloroquine | 30/721 | 4.2 | 1/119 | 0.8 | 0.2 | 0.110 | 31/603 | | 5.1 | 6/136 | | 4.4 | | 0.9 | | 0.725 | **179/1493** | **12.0** | **7/193** | | | **3.6** | | **0.3** | **0.001** |
| SP | **461/721** | **63.9** | **93/119** | **78.2** | **2.0** | **0.003** | **373/603** | | **61.9** | **104/136** | | **76.5** | | **2.0** | | **0.001** | **698/1493** | **46.8** | **138/193** | | | **71.5** | | **2.9** | **< 0.001** |
| Amodiaquine | 86/721 | 11.9 | 8/119 | 6.7 | 0.5 | 0.100 | 69/603 | | 11.4 | 8/136 | | 5.9 | | 0.5 | | 0.060 | **355/1493** | **23.8** | **11/193** | | | **5.7** | | **0.2** | **< 0.001** |
| Quinine | 151/721 | 20.9 | 21/119 | 17.6 | 0.8 | 0.410 | 101/603 | | 16.7 | 14/136 | | 10.3 | | 0.6 | | 0.063 | 116/1493 | 7.8 | 12/193 | | | 6.2 | | 0.8 | 0.445 |
| Artemisin | 11/721 | 1.5 | 0/119 | 0.0 | 0.0 | 0.996 | 43/603 | | 7.1 | 5/136 | | 3.7 | | 0.5 | | 0.147 | 161/1493 | 10.8 | 26/193 | | | 13.5 | | 1.3 | 0.264 |
| Traditional | 1/721 | 0.1 | 0/119 | 0.0 | 0.0 | 0.997 | 2/603 | | 0.3 | 0/136 | | 0 | | 0.0 | | 0.996 | 4/1493 | 0.3 | 0/193 | | | 0 | | 0.0 | 0.996 |

^a^ Complete screening, screening with small holes, glass windows compared to no screening or badly damaged screening

^b^ Complete and partly complete ceiling board compared to no ceiling board
