# Supplementary material for: Microbial Larvicide Application by a Large-Scale, Community-Based Program Reduces Malaria Infection Prevalence in Urban Dar Es Salaam, Tanzania
Source: PLoS One. 2009 Mar 31;4(3):e5107. doi: 10.1371/journal.pone.0005107 (PMC2661378; doi:10.1371/journal.pone.0005107)
Supplement: Box S1 — Malaria prevalence as a function of age and exposure. (0.16 MB DOC) [file pone.0005107.s005.doc]

| ***Box S1. Malaria prevalence as a function of age and exposure***  Initial attempts to examine determinants of malaria prevalence proved difficult to interpret. Original attempts to fit logistic regression models produced counter-intuitive outcomes such as higher social economic status (SES) associated with higher malaria prevalence and high *An. gambiae* densities associated with lower malaria prevalence. We therefore took an in-depth look at possible confounders such as the age-distribution of prevalence, using data from all 15 wards from the year immediately before intervention. Although Dar es Salaam is an urban area with mostly rather low EIR values, the distribution of prevalence across various age groups (Figure SB1.1) was consistent with rural areas where prevalence declines as people get older [1-5]. Overall, malaria prevalence initially appeared to be only very weakly related to locally measured EIR (Figure SB1.2A) when analyzed by GEE treating infection status as a binary dependent variable with a logit link function, controlling for TCU cluster effect, and treating the logarithm of EIR plus 1 as a continuous variable. However, when prevalence was stratified by age, the infection status of young children (0 – 5 years old) showed positive association with EIR (Figure SB1.2B). In contrast, for children over the age of 5, no relationship between prevalence and EIR was observed (Figure SB1.2C). Furthermore, prevalence amongst adults decreases with increasing EIR, presumably due to higher exposure in childhood and therefore elevated levels of acquired immunity (Figure SB1.2D). Overall, this modest but clear peak of prevalence in young children reflects early exposure to infection and development of immunity amongst residents of Dar es Salaam. While such early acquisition of infection and immunity are consistent with reports from rural areas with similarly low transmission levels [2,6-9], the overall prevalence in Dar es Salaam is much lower. This might be explained by faster parasite clearance rates, presumably due to high availability and utilization of curative drugs [10-12] in this urban setting with relatively well developed health services [13-15], possibly augmented by immunity acquired to higher levels of exposure in years preceding this study. | |
| --- | --- |
|  |  |
| **Figure SB1.1 (Above).** The crude age-prevalence profile for malaria infection in TCUs for which EIR estimates are available from year 2 of the study. These are the data analyzed in more detail in SB1.2 below.  **Figure SB1.2 (Left).** Association between malaria prevalence and entomological inoculation rate (EIR) as a function of age. The proportion of residents patently infected in each Ten Cell Unit (TCU) where EIR was also determined is presented as open circles in panels A-D for all ages (A), young children (B), older children and young adults (C) and older adults (D) with best-fit logistic models of prevalence as a function of EIR plotted as continuous lines. The individual human subject was treated as the experimental unit of measurement with TCU identity treated as a cluster effect and the logarithm of the total EIR plus one treated as a continuous variable. |
| **References**  1. Snow RW, Omumbo JA, Lowe B, Molyneaux CS, Obiero JO, et al. (1997) Relation between severe malaria morbidity in children and level of *Plasmodium falciparum* transmission in Africa. Lancet 349: 1650-1654.  2. Marsh K (1992) Malaria-a neglected disease. Parasitology 104 (Supplement): S53-S69.  3. Smith TA, Beck HP, Kitua A, Mwankusye S, Felger I, et al. (1999) Age dependence of the multiplicity of *Plasmodium falciparum* infections and of other malariological indices in an area of high endemicity. Trans R Soc Trop Med Hyg 93 (Supplement 1): 15-20.  4. Molineaux L, Gramiccia G (1980) The Garki Project. Geneva: World Health Organisation. 311 p.  5. Snow RW, Marsh K (2002) The consequences of reducing transmission of *Plasmodium falciparum* in Africa. Adv Parasitol 52: 235-264.  6. Mbogo CN, Snow RW, Khamala CP, Kabiru EW, Ouma JH, et al. (1995) Relationships between *Plasmodium falciparum* transmission by vector populations and the incidence of severe disease at nine sites on the Kenyan coast. Am J Trop Med Hyg 52: 201-206.  7. Mbogo CN, Snow RW, Kabiru EW, Ouma JH, Githure JI, et al. (1993) Low-level *Plasmodium falciparum* transmission and the incidence of severe malaria infections on the Kenyan coast. Am J Trop Med Hyg 49: 245-253.  8. Marsh K, Otoo L, Hayes RJ, Carson DC, Greenwood BM (1989) Antibodies to blood stage antigens of *Plasmodium falciparum* in rural Gambians and their relation to protection against infection. Trans R Soc Trop Med Hyg 83: 293-303.  9. Greenwood BM, Bradley AK, Greenwood AM, Byass P, Jammeh K, et al. (1987) Mortality and morbidity from malaria among children in a rural area of The Gambia, West Africa. Trans R Soc Trop Med Hyg 81: 478-486.  10. Gu W, Mbogo CM, Githure JI, Regens JL, Killeen GF, et al. (2003) Low recovery rates stabilize malaria endemicity in areas of low transmission in coastal Kenya. Acta Trop 86: 71-81.  11. Vercruysse J, Jancloes M, Van de Velden L (1983) Epidemiology of seasonal falciparum malaria in an urban area of Senegal. Bulletin of the World Health Organization 61: 821-831.  12. Gu W, Killeen GF, Mbogo CM, Regens JL, Githure JI, et al. (2003) An individual-based model of *Plasmodium falciparum* malaria transmission on the coast of Kenya. Trans R Soc Trop Med Hyg 97: 43-50.  13. Lorenz N, Mtasiwa D (2004) Health in the urban environment: experience from Dar es Salaam, Tanzania. Ann N Y Acad Sci 1023: 159-163.  14. Few R, Harpam T, Atkinson S (2003) Urban primary health care in Africa: a comparative analysis of city-wide public sector projects in Lusaka and Dar es Salaam. Health Place 9: 45-53.  15. Harpam T, Few R (2002) The Dar es Salaam Urban Health Project, Tanzania: a multi-dimensional evaluation. J Public Health Med 24: 112-119. | |
