# Supplementary material for: Microbial Larvicide Application by a Large-Scale, Community-Based Program Reduces Malaria Infection Prevalence in Urban Dar Es Salaam, Tanzania
Source: PLoS One. 2009 Mar 31;4(3):e5107. doi: 10.1371/journal.pone.0005107 (PMC2661378; doi:10.1371/journal.pone.0005107)
Supplement: Box S2 — Contributions of interventions other than larviciding to malaria prevention. (0.05 MB DOC) [file pone.0005107.s006.doc]

| **Box S2. Contributions of interventions other than larviciding to malaria prevention**  Examining time trends for the use of protective measures and drugs over these 3 years, overall ITN usage remained consistently low but window screening and ceiling boards became increasingly common (Figure SB2.1). Interestingly, the use of both amodiaquine and artemisin-based drugs increased while the use of quinine and sulphadoxine-pyrimethamine decreased significantly over the three years. Although usage of artemisinin-based therapies increased slightly over the three years of the study, this treatment option remained a remarkably infrequent choice. We attribute poor uptake of this high priority intervention to lack of affordable, subsidized drugs at public facilities until early 2007 and the predominant reliance upon private sector outlets amongst Dar es Salaam residents [1]. Indeed the phasing out of sulphadoxine-pyrimethamine seems to have resulted in higher use of amodiaquine rather than artemisinin-based therapies. These modest increases in the use of effective drugs, perhaps combined with increasing use of screening and complete ceilings, may well have played a role in the overall reduction of malaria prevalence over these three years.  **Reference.**   1. Wang SJ, Lengeler C, Mtasiwa D, Mshana T, Manane L, et al. (2006) Rapid urban malaria appraisal (RUMA) II: Epidemiology of urban malaria in Dar es Salaam (Tanzania). Malar J 5: 29. |
| --- |
|  |
| **Figure SB2.1.** Time trends of protective measures and drug use in the survey areas of the Urban Malaria Control Program. The overall trends over time were calculated using a logistic regression model with the protection measures and drugs as an outcome. Except for ITN usage (P = 0.507), usage of other protective measures and drugs all significantly increased or decreased (P < 0.001). |
